# Unravelling the Drivers of Early-Life Mortality in Yak Calves: A Retrospective Cohort Study Using Survival Analysis

**DOI:** 10.3390/ani16162614

**Published:** 2026-08-20

**Authors:** Asish Debbarma, Mokhtar Hussain, Martina Pukhrambam, Shubham Loat, Vijay Paul, Dinamani Medhi, Sayed Nabil Abedin, Mihir Sarkar

**Affiliations:** 1 ICAR-National Research Centre on Yak, Dirang 790101, Arunachal Pradesh, India; mokhtar.hussain@icar.org.in (M.H.); martina.pukhrambam@icar.org.in (M.P.); shubham.loat@icar.org.in (S.L.); vijay.paul@icar.org.in (V.P.); dinamani.medhi@icar.org.in (D.M.); director-nrcy@icar.org.in (M.S.); 2 ICAR-Indian Veterinary Research Institute, Mukhteswar Campus, Nainital 263138, Uttarakhand, India; sayed.abedin@icar.org.in

**Keywords:** yak calf, mortality, survival analysis, Kaplan–Meier estimator, Cox proportional hazards, retrospective study, risk factors

## Abstract

Early-life calf mortality is a significant barrier to herd growth and overall productivity in yak husbandry. This retrospective study investigated the survival pattern and potential risk factors linked to yak calf mortality during the first 90 days of life. Analysis was performed on existing farm records from 428 yak calves born between 2013 and 2025 at a semi-intensive yak farm. The Kaplan–Meier method was used to estimate survival probability, and the log-rank test was used to compare survival probabilities; additionally, a multivariate Cox proportional hazards model was employed to identify independent risk factors. Overall, 70 (16.4%) calves died within the first 90 days after birth. The Kaplan–Meier analysis indicated significantly lower survival probability among low-birth-weight calves (≤12 kg; *p* = 0.0005) and calves born to primiparous dams (*p* = 0.0003). In contrast, survival did not differ according to calf sex (*p* = 0.620) and birth season (*p* = 0.658). In the multivariate Cox proportional hazards model, low birth weight (*HR* = 2.27, 95% *CI*: 1.24–4.16; *p* = 0.008) and primiparous dams (*HR* = 2.08, 95% *CI*: 1.28–3.38; *p* = 0.003) were independently associated with higher mortality risk. As a result, calf sex and birth season were not significant predictors. Diarrhea and respiratory disease were the predominant causes of calf deaths. The current study’s results indicate that maternal and neonatal factors are the major determinants of early-life calf survival. This highlights the need for special care with optimum nutrition and regular monitoring of primiparous dams and low-birth-weight calves in order to lower pre-weaning mortality and improve overall herd productivity.

## Simple Summary

Improving calf survival is essential for enhancing productivity and sustainability in high-altitude yak production. However, information on the critical determinants influencing yak calf mortality remains scarce. Using a 13-year retrospective cohort, this study evaluated survival patterns and risk factors associated with calf mortality during the first 90 days of life. Low birth weight and primiparous dams were identified as the major determinants of calf mortality using the Kaplan–Meier estimator and a multivariate Cox proportional hazards model, whereas calf sex and birth season did not affect survival. Diarrhea and respiratory disease were the leading causes of death in yak calves. Our study provides practical evidence for prioritizing high-risk calves and first-parity dams, thereby contributing to improving calf survival and sustainable yak production.

## 1. Introduction

Yaks (*Bos grunniens*), unique highland herbivores that are well adapted to high altitudes between 3000 and 5000 m above sea level, are mostly concentrated in the Qinghai–Tibetan Plateau (QTP) and across Himalayan regions [1,2]. At present, there are approximately 14.2 million domesticated yaks, of which 95% are prevalent in China, but they are also found in neighboring countries including India, Nepal, Bhutan, Mongolia, Russia, Pakistan, Afghanistan, and Tajikistan [3,4]. Yaks are exceptionally adapted to adverse agro-climatic conditions characterized by severe cold, hypoxia, and seasonal feed scarcity, which cannot sustain other types of livestock efficiently [5]. In India, yaks are primarily raised in high-altitude regions of Arunachal Pradesh, Sikkim, Himachal Pradesh, and Ladakh. This species is associated with livelihood security, socio-economic growth, cultural identity, and overall ecosystem stability [6]. They provide valuable products, including milk, meat, skin, wool, and dung, to local tribal communities. Additionally, this ruminant is used as a draught power in agriculture, transport, and tourism sectors and is equally important in religious and ethnic traditional ceremonies for pastoralists [7].

Calf mortality is a vital marker for herd health, managerial efficiency, and animal welfare in sustainable yak farming [8]. Over half of calf deaths occur within the first seven to ten days after birth in livestock production systems [9]. Furthermore, the highest risk of death occurs during the first four weeks of the calf’s life [10]. Mortality during the neonatal and pre-weaning stages results in significant economic losses, leading to delayed genetic progress and substantially lower availability of calves for replacement; additionally, it incurs production and management costs. In bovids, delayed colostrum intake, failure of transfer of passive immunity (FTPI), dystocia, environmental stress, and poor management practices are considered essential risk factors for calf mortality [11,12,13]. In yak calves, neonatal survival is further challenged by the extreme environmental conditions of being raised in high-altitude regions, including hypoxia, severe cold stress, feed scarcity, and rugged and difficult terrain [4,5,14].

Previous findings on yak calf mortality indicate that a significant number of deaths occur within the first 3 months of life. Interestingly, an earlier study by our institute reported that approximately 37% of yak calves died within the first 30 days of life [15]. Furthermore, they reported higher calf mortality during summer than winter, which was attributed to seasonal environmental conditions, particularly higher temperature, humidity, and rainfall, which may exacerbate respiratory and digestive disorders and increase disease pressure during the rainy season [15]. Additionally, the major diseases affecting calf mortality were diarrhea, pneumonia, parasitic infestations (e.g., *Toxocara vitulorum*, *Strongyle* spp., *Trichuris* spp., and *Eimeria* spp.), navel infections, and other gastrointestinal disorders [16,17]. Survival analysis provides a comprehensive statistical framework and advantages over cross-sectional studies since it evaluates the chances of survival over time and determines risk factors impacting mortality dynamics [18]. The Kaplan–Meier estimator is widely recognized as a reliable method for estimating survival. Additionally, Cox proportional hazards analysis has been widely used to evaluate the hazard ratios associated with dairy calf mortality [19,20,21]. Evidence from dairy cattle suggests that the combined influence of birth weight and dam parity may affect neonatal survival [22], but this relationship has not been adequately evaluated in highland yak exposed to the distinctive physiological and environmental challenges of high-altitude production systems. To our knowledge, such a predictive approach has rarely been applied to yak calf survival.

The first three months of life represent a critical period for calf survival, as passive immunity progressively declines while the adaptive immune system is still developing, increasing susceptibility to enteric and respiratory diseases. Previous studies in bovine dairy calves have similarly identified this early-life period as important for evaluating survival and identifying host- and management-related risk factors associated with mortality [23,24]. Therefore, the present study used a 13-year retrospective farm registry dataset to analyze survival probability and hazard of mortality in yak calves during the first 90 days of life and also to determine the potential risk factors associated with calf mortality. This study addresses a core need in yak veterinary epidemiology and would act as baseline evidence-based knowledge to assist herd health management for high-altitude yak production systems.

## 2. Materials and Methods

This study was a retrospective cohort analysis from a relatively small herd-size semi-intensive yak farm of ICAR–National Research Centre on Yak, Dirang, Arunachal Pradesh, India, situated at Nyukmadung, located at an altitude of 9000 ft above sea level (geographical coordinates: 27°25′ N and 92°07′ E). The region has a sub-temperate climate, with summer temperatures generally ranging from 20 to 30 °C and winter temperatures from −3 to 12 °C. Mean annual rainfall is approximately 1704.80 mm, with reported annual precipitation ranging from approximately 1560 to 2500 mm, and with most of the rainfall occurring during the monsoon period. The farm maintains a pure breed of *Arunachali* Yak (Accession no.: INDIA_YAK_2300_ARUNACHALI_16001). The study herd was maintained under a semi-intensive production system. Adult yaks were grazed on natural pasture during the day, except during periods of heavy rainfall when grazing was restricted, and were supplemented twice daily (09:00 and 15:00 h) with concentrate feed, fresh green fodder, and ad libitum access to clean drinking water, as per ICAR feeding systems [25]. In the institute, female yaks were first bred artificially at a mean age of 3.1 ± 0.2 years, or after attaining a mean body weight of 242.4 ± 11.6 kg. Artificial insemination was conducted throughout the year, with females bred according to their reproductive status rather than being restricted to a defined breeding season. Following birth, calves remained with their dams and were allowed to suckle freely and accompany them during grazing, except on rainy days when both dams and calves were housed together. Additionally, yak calves were generally observed to initiate suckling within approximately 30 min post-partum under the supervision of a farm attendant. Due to strong maternal instinct, yak calves were weaned at 8 months of age in accordance with the farm’s routine management protocol. All the animals were maintained according to standard farm management practices.

The data were collected and retrieved from the farm registry over a span of 13 years, from 2013 to 2025. The data include live calf birth, date of birth, sex, age, dam parity, birth weight (BW), birth season, disease occurrence, age at death (survival time), and cause of death. As this was a retrospective cohort study spanning 13 years (2013–2025), the total herd size and the number of breeding females were not constant but varied annually due to births, culling, mortality, and routine herd replacement (Supplementary Table S1). Therefore, a single herd size is not representative of the entire study period. A breeding ratio of approximately 1 breeding bull to 30 breeding females was maintained for semen collection and breeding management at the farm. The survival analysis included all yak calves born during the study period that met the study inclusion criteria.

The primary outcome was time to death from birth, measured in days. The survival endpoint was defined as 90 days after birth. Calves that died within 90 days of birth were considered events (mortality = 1). Calves that survived to 90 days or were no longer available for continued observation were treated as right-censored observations (mortality = 0) at their last recorded observation. Calves that died within the first 90 days of life were treated as events (mortality = 1). Calves that survived beyond 90 days or were no longer available for continued observation were treated as censored (mortality = 0) at their last recorded date. Because this was a retrospective study based on routine farm records, the specific reasons for censoring (e.g., remaining alive, sale, or loss to follow-up) were not consistently documented throughout the study period. The main predictor variables include: birth weight (≤12 kg or >12 kg), sex (male/female), dam parity (primiparous/multiparous), birth season (winter = November–February; summer = March–June; rainy = July–October). In the present study, birth weight was categorized into low birth weight (≤12 kg) and high birth weight (>12 kg). This threshold was selected based on the birth-weight distribution and characteristics of the present study population, as no universally accepted biological cut-off for low birth weight is established for yak calves. Furthermore, birth weight was also evaluated as a continuous variable to confirm model robustness and prevent the loss of statistical information. Yak calves have substantially lower birth weights than purebred cattle breeds, where the average birth weights of yak calves generally range between 11 and 13 kg [26]. In addition, this cut-off was consistent with the distribution of birth weights observed in the present cohort and enabled the identification of calves at increased risk of early-life mortality. Data on the cause of death were obtained from the farm mortality and treatment register for all calves that died within the first 90 days of life. Causes of mortality were classified as diarrhea, bovine respiratory disease (BRD), tympany, and other causes according to the clinical diagnosis documented in the farm registry.

Descriptive statistics were used to summarize baseline characteristics and calf mortality patterns. Survival probability was determined using the Kaplan–Meier survival method. Survival curves were generated for different categories and evaluated by the log-rank test. Subsequently, a multivariate Cox proportional hazards model was fitted to identify potential predictors associated with calf mortality. To determine whether the association between birth weight and 90-day calf mortality varied according to dam parity, a biologically plausible multiplicative interaction term (birth weight × dam parity) was evaluated by including it in the multivariable Cox proportional hazards frailty model. The statistical significance of the interaction was assessed using the Wald test. Interaction terms that were not statistically significant were not retained in the final model. Subsequently, a multivariable Cox proportional hazards regression model incorporating all biologically relevant variables was fitted to identify independent predictors of calf mortality. Birth year was included as a fixed effect in the multivariable Cox model to account for temporal variation during the 2013–2025 study period. The proportional hazards assumption of the Cox model was evaluated using scaled Schoenfeld residuals with the cox.zph function in the R survival package. Both global and covariate-specific proportional hazards tests were performed, and scaled Schoenfeld residual plots were visually inspected to assess whether regression coefficients remained constant over time. A *p*-value greater than 0.05 was considered to indicate no evidence against the proportional hazards assumption. The Cox proportional hazards model expresses the hazard of calf mortality at time t as the product of an unspecified baseline hazard function and an exponential function of the covariates. Hazard ratios (HRs) with corresponding 95% confidence intervals (CIs) were estimated to quantify the association between each predictor variable and the risk of mortality while adjusting for the remaining covariates included in the model.

To account for potential non-independence of observations arising from repeated births from the same dams across the 13-year study period, a shared frailty model (incorporating individual Dam ID as a random effect) was evaluated alongside the fixed-effects Cox proportional hazards model.

Statistical differences were considered at *p* < 0.05. All statistical analyses were performed using R statistical software (R version 4.6.0, 2026), and figures were prepared using RStudio Desktop (version 2024.12.1+563; Posit Software, PBC, Boston, MA, USA).

The study was conducted in accordance with institute guidelines for the use of retrospective datasets, and no ethical approval was needed since it involved no experimental procedures on animals.

## 3. Results

### 3.1. Descriptive Characteristics

A total of 428 yak calves born between 2013 and 2025 were included in the present study (Table 1). During the study period, 70 mortality events were recorded, indicating an overall mortality rate of 16.4%, whereas 358 calves survived beyond the first 90 days of life. Out of the total number of calves, 221 (51.6%) were male and 207 (48.4%) were female. Based on birth weight, 42 calves (9.8%) were categorized as low birth weight (≤12 kg), while 386 calves (90.2%) were categorized as high birth weight (>12 kg). The percentage of low-BW calves (≤12 kg) was substantially higher among calves that died (20.0%) compared to the survivors (7.8%; *p* = 0.003). Furthermore, male yak calves had a significantly higher birth weight than female calves (15.73 ± 3.60 vs. 15.00 ± 3.78 kg; mean ± SD; Welch’s *t*-test, *p* = 0.043). Dam parity was also significantly associated with calf mortality. Calves born to primiparous dams exhibited a mortality rate of 26.9%, which was more than two-fold higher than the mortality rate observed in calves born to multiparous dams (11.0%; *p* < 0.001). No significant differences in calf mortality were observed according to calf sex (*p* = 0.723) or birth season (*p* = 0.658).

### 3.2. Kaplan–Meier Survival Curves

**Figure 1:**
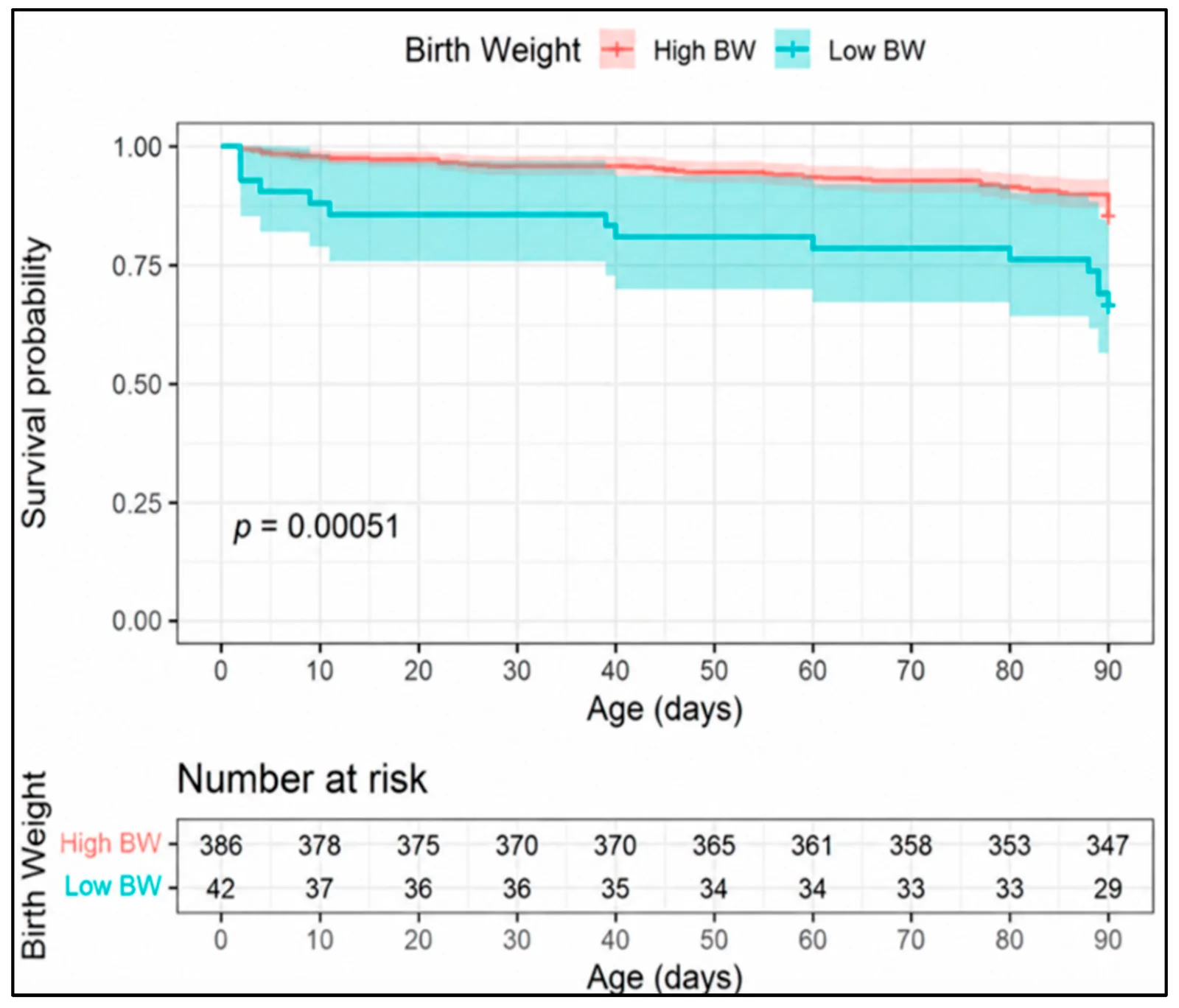
Kaplan–Meier survival curves showing the probability of survival of yak calves by birth weight category up to 90 days of age. Calves were classified as low BW (≤12 kg) and high BW (>12 kg). Shaded areas represent 95% confidence intervals. Censored observations occurred predominantly at the administrative end of the 90-day follow-up period; therefore, the censoring marks overlap near the end of the curves and are not individually distinguishable.

Based on the Kaplan–Meier survival analysis, our findings showed a significant effect of birth weight on calf survival during the first 90 days of life (Figure 1). The estimated overall Kaplan–Meier survival probabilities at 7, 30, 60, and 90 days were 97.4% (95% *CI*: 95.9–98.9%), 94.9% (95% *CI*: 92.8–97.0%), 92.1% (95% *CI*: 89.5–94.7%), and 83.6% (95% *CI*: 80.2–87.2%), respectively (Supplementary Table S3). The median survival time was not estimable because the overall survival probability remained above 50% throughout the 90-day follow-up period. Calves with low BW (≤12 kg) displayed a significantly lower survival probability during subsequent observation than calves with high BW (>12 kg). The differences between the survival curves became noticeable within the first few weeks after birth (i.e., the early neonatal period) and remained until the endpoint. Additionally, lower-BW calves experienced a steeper decline in survival probability. By 90 days, survival probability was visibly lower in the low-BW category than the high-BW category. The estimated mean survival time was 74.7 days for calves in the low-birth-weight group compared with 85.5 days for those in the high-birth-weight group, with surviving calves treated as right-censored observations (log-rank χ^2^ = 12.085, *p* = 0.0005).

**Figure 2:**
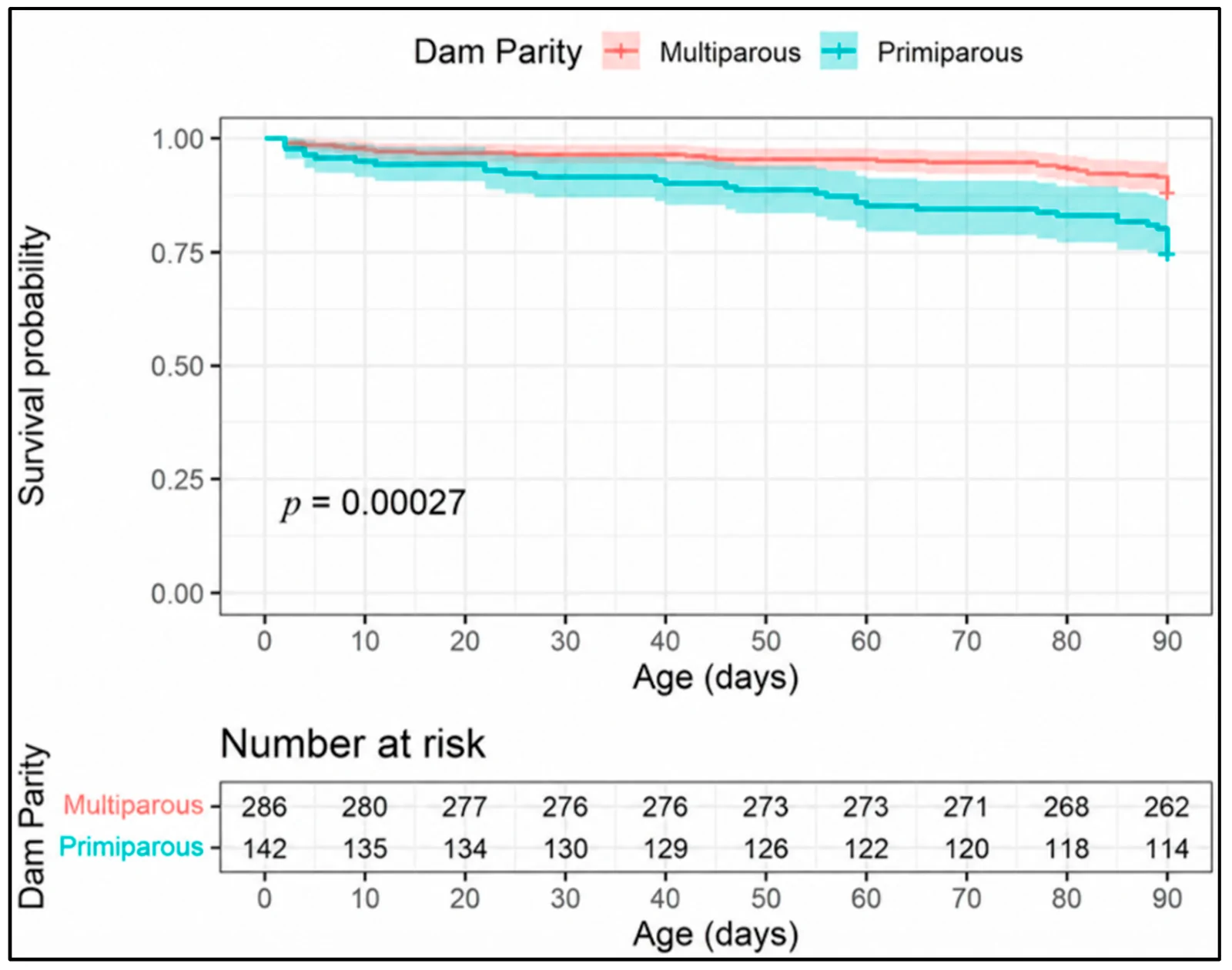
Kaplan–Meier survival curves showing the probability of survival of yak calves until 90 days of age based on dam parity. Calves were grouped as born to primiparous (parity = 1) or multiparous (parity > 1) dams. Shaded areas represent 95% confidence intervals. Censored observations occurred predominantly at the administrative end of the 90-day follow-up period; therefore, the censoring marks overlap near the end of the curves and are not individually distinguishable.

Similarly, survival probabilities differed significantly according to dam parity (Figure 2). Calves born to primiparous dams had lower survival rates during the study period than calves born to multiparous dams. The survival curves began to diverge within the first 10 days of life, with lower survival among calves born to primiparous dams, and this separation persisted throughout the 90-day observation period. The difference between the survival distributions was statistically significant (χ^2^ = 13.237, *p* = 0.0003).

**Figure 3:**
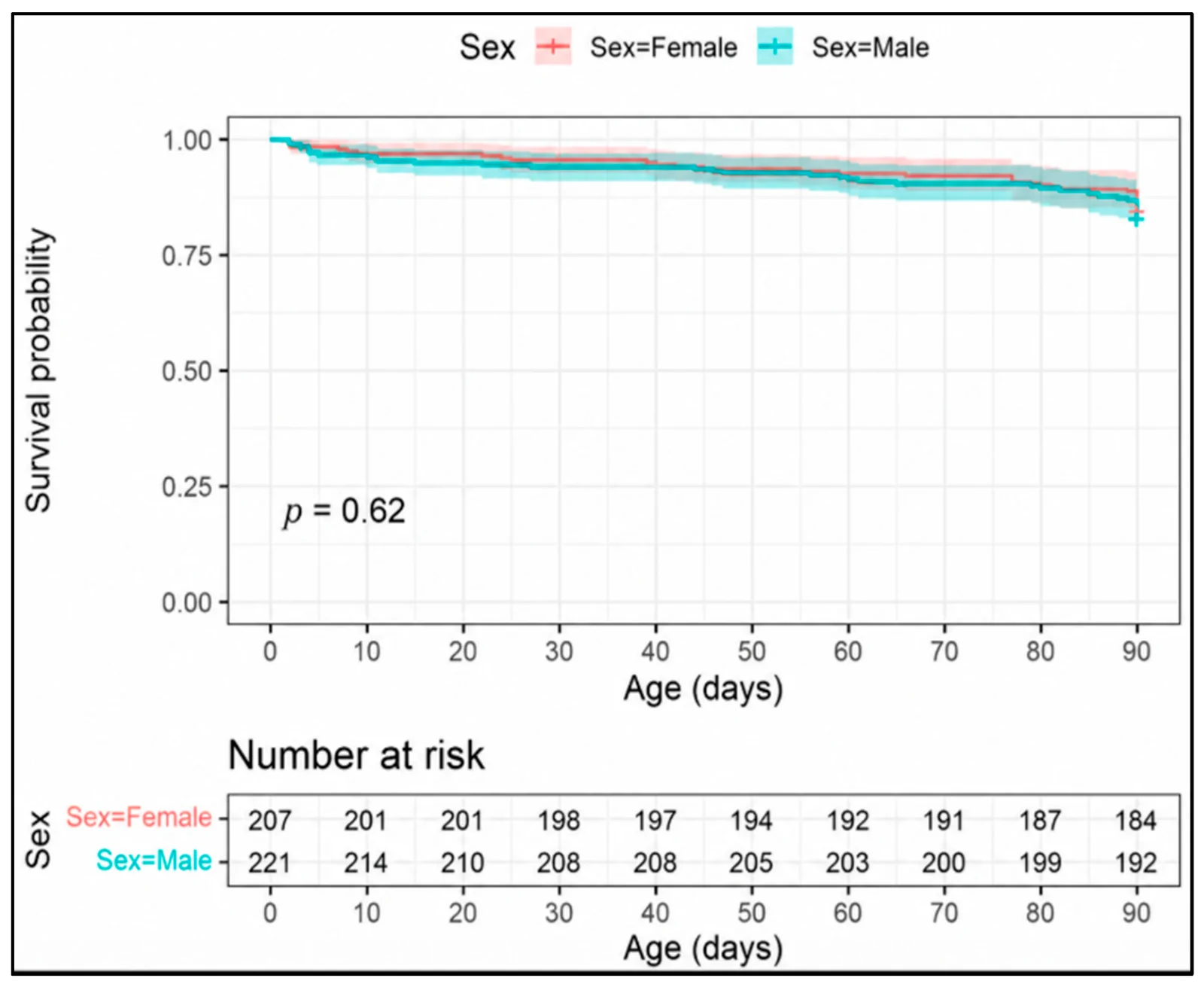
Kaplan–Meier survival curves showing the probability of survival of yak calves until 90 days of age based on sex. Shaded areas represent 95% confidence intervals. Censored observations occurred predominantly at the administrative end of the 90-day follow-up period; therefore, the censoring marks overlap near the end of the curves and are not individually distinguishable.

Survival probability did not differ significantly between male and female calves (Figure 3; log-rank χ^2^ = 0.25, *p* = 0.616).

### 3.3. Cox Proportional Hazards Analysis

A multivariable Cox proportional hazards regression model was used to identify independent risk factors linked to 90-day mortality of yak calves (Table 2). The multivariable model included all pre-specified biologically relevant variables (birth weight, dam parity, calf sex, birth season, and birth year), irrespective of their univariable statistical significance. Calf mortality was found to be significantly correlated with low birth weight and dam parity. Calves with low BW (≤12 kg) exhibited a 2.27-fold higher risk of mortality than calves weighing >12 kg at birth (*HR* = 2.27, 95% *CI* = 1.24–4.16; *p* = 0.008). When analyzed as a continuous predictor, birth weight remained significantly associated with reduced 90-day mortality risk, with each 1 kg increase in birth weight decreasing the hazard of mortality by approximately 16% (*HR* = 0.84, 95% *CI*: 0.78–0.90, *p* < 0.001). Calves born to primiparous dams had a 2.08 times higher hazard of mortality compared with calves of multiparous dams (*HR* = 2.08, 95% *CI* = 1.28–3.38; *p* = 0.003).

**Figure 4:**
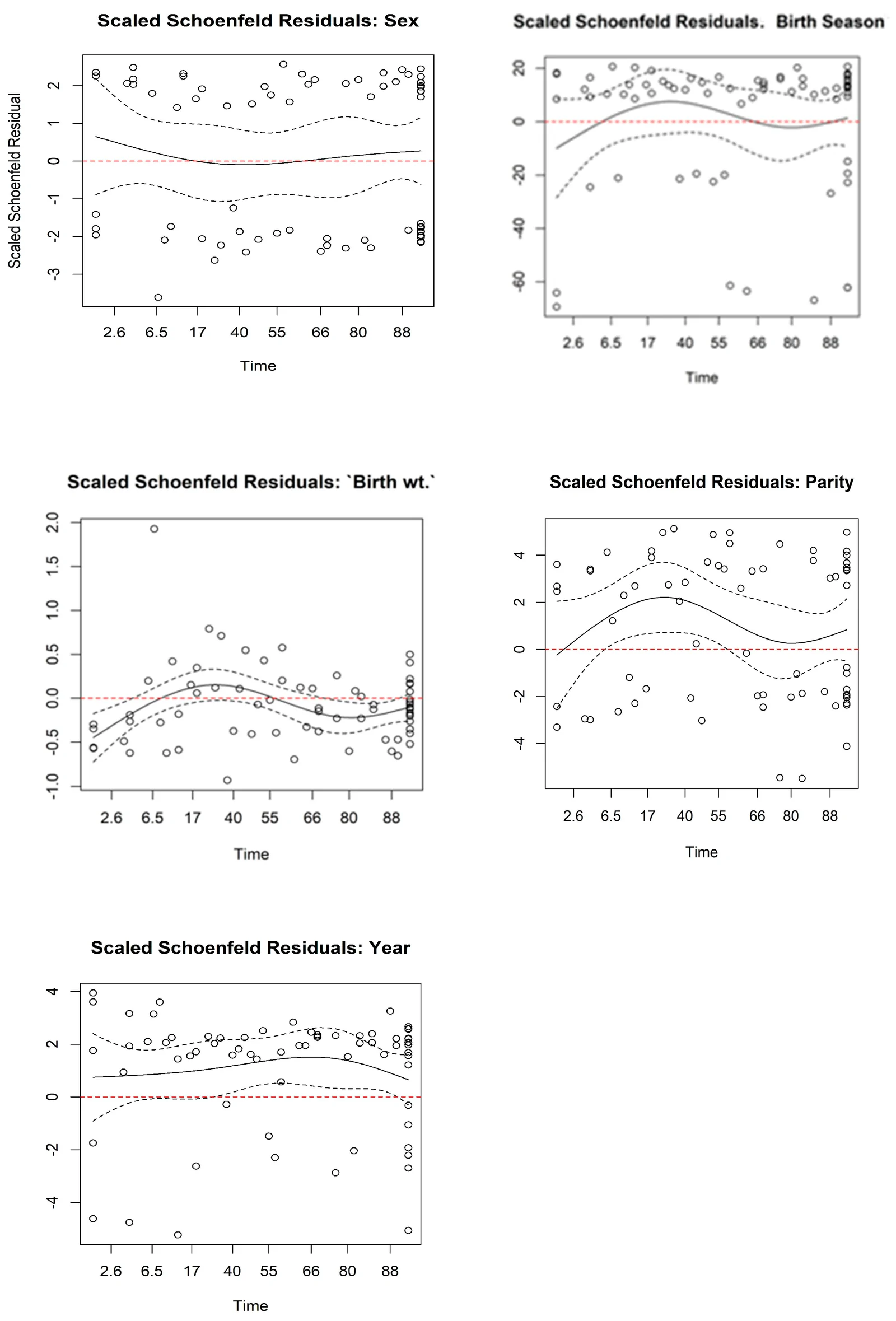
Scaled Schoenfeld residual plots for the covariates included in the multivariable Cox proportional hazards model: birth weight, dam parity, calf sex, birth season, and birth year. The solid line represents the smoothed estimate of the time-varying regression coefficient, while the dashed lines indicate approximate 95% confidence limits.

In contrast, calf sex and birth season were not strongly associated with mortality risk. Male calves exhibited a mortality hazard similar to that of female calves (*HR* = 1.23, 95% *CI* = 0.76–1.98; *p* = 0.398). Subsequently, neither summer-born calves (*HR* = 0.86, 95% *CI* = 0.39–1.90; *p* = 0.701) nor winter-born calves (*HR* = 0.80, 95% *CI* = 0.41–1.57; *p* = 0.515) differed substantially in mortality risk from calves born during the rainy season. The fitted Cox proportional hazards model demonstrated acceptable discriminatory ability with a concordance index (C-index) of 0.700 (SE = 0.030). Overall model significance was supported by the likelihood ratio test (χ^2^ = 38.31, df = 17, *p* = 0.002), the Wald test (χ^2^ = 36.97, df = 17, *p* = 0.003), and the score (log-rank) test (χ^2^ = 40.65, df = 17, *p* = 0.001), indicating good overall model performance. To account for the potential non-independence arising from repeated births from the same dams during the study period, a shared frailty Cox proportional hazards model incorporating Dam ID as a random effect was additionally fitted. The frailty analysis yielded results that were highly consistent with those of the primary multivariable Cox proportional hazards model, confirming that low birth weight and primiparous dam remained significant independent predictors of 90-day calf mortality (Supplementary Table S2). Assessment of Schoenfeld residuals revealed no evidence of violation of the proportional hazards assumption, either globally (χ^2^ = 29.33, df = 23, *p* = 0.170) or for any individual covariate. Visual inspection of the scaled Schoenfeld residual plots supported these findings. Similarly, none of the individual covariates showed evidence of non-proportional hazards, including sex (*p* = 0.963), birth season (*p* = 0.953), birth weight (*p* = 0.884), parity (*p* = 0.260), and birth year (*p* = 0.063), as shown in Table 3. Visual inspection of the scaled Schoenfeld residual plots also revealed no systematic temporal trends in the regression coefficients, supporting the suitability of the Cox proportional hazards model for the present analysis (Figure 4). Evaluation of the birth weight × dam parity interaction showed no statistically significant interaction (*HR* = 2.42, 95% *CI*: 0.55–10.65, *p* = 0.242), indicating that birth weight and dam parity acted as independent predictors of calf mortality (Supplementary Table S4). Therefore, only the main effects were retained in the final multivariable model.

### 3.4. Causes of Calf Mortality

**Figure 5:**
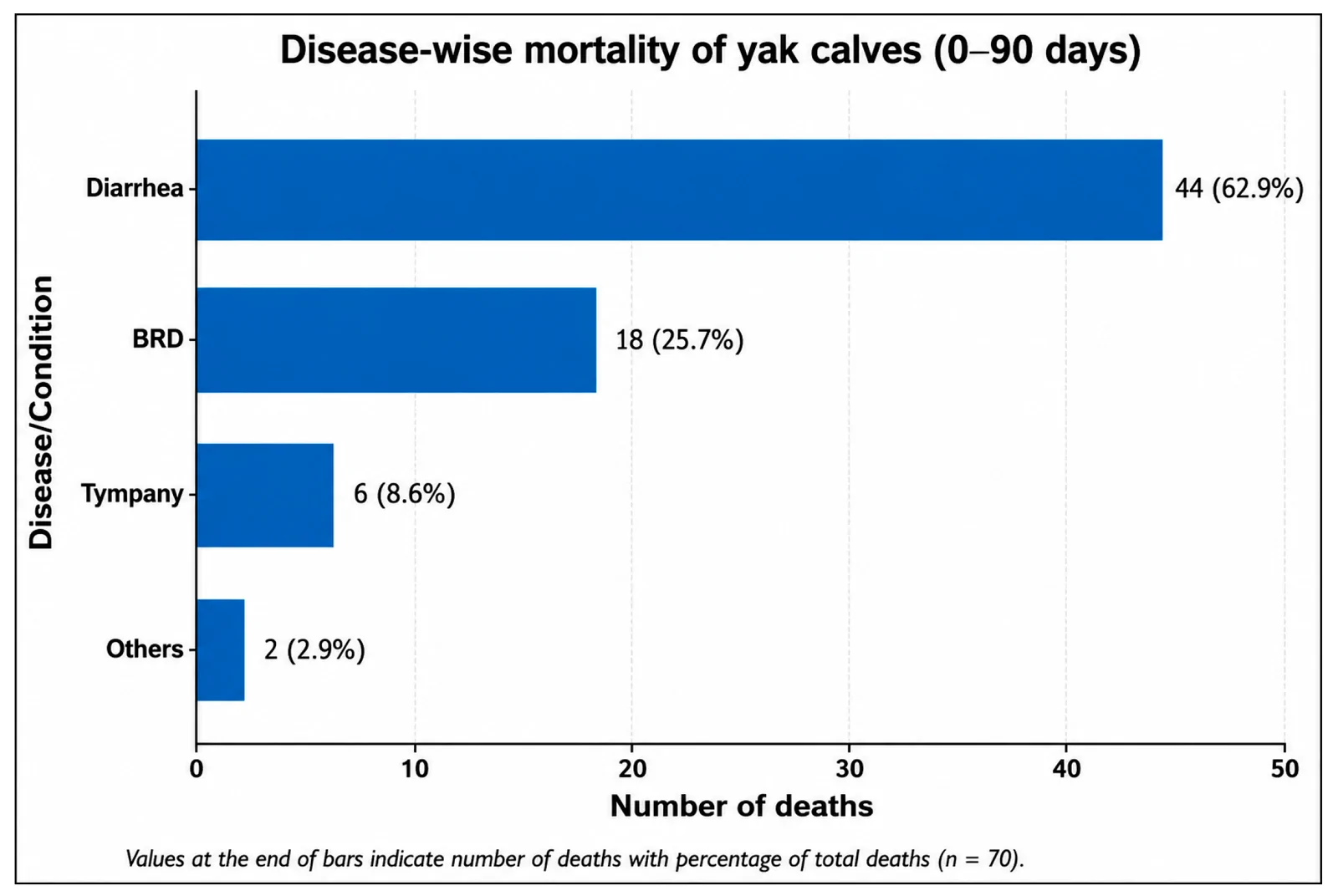
Distribution of causes of mortality among yak calves during the first 90 days after birth. Values at the end of each bar indicate the number of deaths with corresponding % of total deaths (*n* = 70).

The distribution of causes of mortality among yak calves that died during the first 90 days of life is presented in Figure 5. The present study showed that diarrhea was the major cause of mortality, accounting for 62.9% of deaths, followed by bovine respiratory diseases (BRD; 25.7%). Tympany contributed to 8.6% of deaths, whereas other unknown causes accounted for only 2.9%. The findings indicate that gastrointestinal and respiratory disorders in combination account for more than 90% of yak calf mortality.

## 4. Discussion

The present study is one of the few long-term retrospective studies that have used survival analysis methods to assess calf survival dynamics and potential risk factors associated with pre-weaning mortality in yak calves. The current study showed that birth weight and dam parity are the two important factors influencing calf survival during the first 90 days of life by evaluating 428 yak calves born between 2013 and 2025. K-M survival analysis showed significantly lower survival rates among low-birth-weight calves and calves born to primiparous dams. Subsequently, both factors independently affected the mortality risk, which was confirmed by the multivariable Cox proportional hazards model. Furthermore, evaluation of the birth weight × dam parity interaction demonstrated no statistically significant interaction between these variables, indicating that birth weight and dam parity acted as independent rather than synergistic determinants of early-life mortality. Conversely, no significant correlations were observed among calf sex, birth season, and mortality risk. The present findings suggest that neonatal and maternal factors are the two important determinants for early-life survival in yak production systems.

Globally, calf mortality is widely considered one of the major constraints in livestock production due to a reduction in replacement stock, hindering genetic development and reducing overall herd productivity [21,23]. In yak husbandry, similar observations have been documented [1], and it has been pointed out that calf mortality is a significant problem in high-altitude ecosystems characterized by severe climatic conditions, feed scarcity, and inaccessibility to veterinary services. Birth weight has been identified as the most reliable indicator of calf survival [27]. In the present study, calves weighing ≤12 kg at birth demonstrated significantly lower survival probabilities and a 2.27-fold greater hazard of mortality than calves with high birth weight (>12 kg). The present findings concur with previous reports on large ruminants, which have identified low birth weight as a strong predictor of neonatal mortality and decreased early-life survival [28]. The greater mortality rate reported in low-birth-weight calves is biologically justifiable, as birth weight reflects prenatal growth and neonatal physiological maturity. Low-birth-weight calves may have fewer energy reserves and less resilience throughout the early postnatal period, making them more vulnerable to sickness and mortality. Low birth weight may be a potential marker of impaired fetal growth and reduced neonatal vigor; therefore, our findings indicate that low birth weight is a strong predictor of early-life survival in yak calves. Low-birth-weight calves may have lower metabolic reserves, poorer thermoregulation, and reduced colostrum intake, potentially increasing their susceptibility to environmental stressors, including hypothermia, infectious diseases, and early mortality [29,30,31]. Although impaired thermoregulation, reduced colostrum intake, and inadequate passive immune transfer have been suggested as possible causes in neonatal ruminants, these variables were not evaluated in the current study and should, therefore, be considered plausible hypotheses requiring confirmation in future prospective investigations. Our results showed that lower birth weight considerably increases the hazard ratio for mortality, highlighting its important role in the neonatal survival of yak calves [32]. This is in line with previous studies in yak species, where high birth weight corresponded to improved physiological resilience and oxidative tolerance [33]. The ≤12 kg cut-off for the body weight threshold should be interpreted as a cohort-specific classification derived from the characteristics of the present study population, rather than a universally applicable biological threshold for yak calves. Although maternal body weight and nutritional status are recognized determinants of calf birth weight, these variables were not consistently available in the farm registry records. Nevertheless, dam parity—an important maternal characteristic reflecting reproductive maturity and maternal experience—was included in the present analysis and was identified as a significant predictor of calf survival. Future prospective studies incorporating detailed maternal phenotypic and nutritional measurements would further improve the understanding of factors influencing birth weight and neonatal survival in yak calves. The proposed physiological mechanisms should, therefore, be interpreted as biologically plausible hypotheses supported by the previous literature rather than findings directly demonstrated by the present retrospective study.

Many earlier studies have demonstrated that calves born to primiparous cows have a greater risk of pre-weaning mortality than calves born to multiparous dams [24,28,34,35,36,37]. Similarly, our findings on dam parity demonstrated that calves born to primiparous dams had a significantly higher risk of mortality. This is consistent with previous reports of increased susceptibility to perinatal problems reported in other bovine species [28], where the authors reported that primiparous Holstein cows had a 2.4-fold higher risk of perinatal mortality, which subsequently increases the chances of developing dystocia 4.7-fold compared to multiparous cows. The higher mortality risk among calves born to primiparous dams may be attributable to inexperienced maternal behavior, poor dam–calf bonding, a low quality and quantity of colostrum, and smaller placental mass—all of which are likely to influence early-life calf survival [23,38,39]. Furthermore, it was observed that the colostrum IgG concentrations of primiparous dams were significantly lower than those of multiparous dams [40]. Hence, these factors may partly explain the higher mortality risk observed among calves born to primiparous dams. Therefore, the strong maternal parity effect observed in the present study suggests that primiparous dams require improved nutritional management during gestation and appropriate neonatal calf care to reduce the susceptibility of yak calves to early-life mortality [13,41]. Another limitation of the present retrospective study was the lack of information on maternal body weight and body condition during gestation [22]. These maternal characteristics are known to influence fetal growth and neonatal birth weight [27], but they were not consistently recorded in the farm records and, therefore, could not be evaluated. Similarly, maternal behavior, colostrum yield, and passive immune transfer were not assessed in the present retrospective study, and therefore these mechanisms remain speculative. Although dam parity was included as an indicator of maternal effects, future prospective studies incorporating maternal body weight, body condition, and nutritional status would provide a more comprehensive understanding of the determinants of birth weight and early-life survival in yak calves.

In contrast to birth weight and maternal parity, calf sex did not significantly influence survival probability. Yaks are well adapted to high-altitude conditions and possess similar physiological adaptation traits; hence, environmental stress is unlikely to affect one sex more than the other [1]. Researchers have shown that sex was not identified as an independent potential indicator of calf mortality in intensive and pasture-based dairy farming systems [24,42,43]. The present findings demonstrate that male calves tend to exhibit a higher hazard risk than female calves; however, this was not statistically significant. This lack of association is consistent with the earlier literature, where the impact of calf sex remains unclear, with the majority of studies reporting that no apparent impact is noticeable on early-life survival rates [44]. A previous study of our institute reported that male calves are more vulnerable to neonatal mortality due to lower serum immunoglobulins, which are required for passive immunity and calf survival [15]. For instance, it was observed that female calves had higher odds of survival compared with male calves born to multiparous dams [45]. Therefore, our analysis suggests that calf sex provides lower predictive significance for survival than the combined effect of maternal and neonatal factors, such as birth weight and dam parity, on the survival probability of yak calves.

Regarding birth season, our analysis showed that it had no statistical significance on yak calf survival. This lack of seasonal fluctuation likely indicates the species’ restricted reproductive adaptation to a highly synchronized calving period since it is a seasonal breeder, which coincides with the short parturition period in spring and early summer [46]. Additionally, the adaptive traits of yak in harsh, high-altitude environments are consistent with the present findings [1]. In contrast, seasonal variation has been identified as a major determinant of calf mortality in other ruminant species [23]. Nevertheless, consistent with our findings, it has been observed that season has no role in calf mortality [47]. The absence of seasonal differences in birth weight or calf survival may reflect the relatively uniform herd management practiced at the institute, where pregnant females received consistent nutritional supplementation throughout the year under a semi-intensive production system.

Diarrhea was the leading cause of mortality in yak calves in the present study, followed by respiratory diseases. However, because this study was purely based on retrospective farm records, the specific etiological agents responsible for diarrhoeal disease were not identified. In neonatal bovids, diarrhoea is mostly multifactorial and may result from bacterial, viral, or protozoal enteric infections, often in combination with inadequate passive transfer, environmental contamination, and management-related stress [30]. The higher incidence of diarrhea observed in the present study indicates a substantial failure in neonatal gut immunity, where inadequate passive transfer of immunoglobulins increases susceptibility to enteric pathogens. This is consistent with previous findings that pointed out that diarrhea and pneumonia were the major causes of calf mortality [45]. Similarly, in intensive dairy farms, neonatal diarrhea and respiratory diseases are considered the primary causes of pre-weaning mortality [23,24]. However, in free-range grazing systems, it was reported that wildlife depredation was the major cause of yak mortality, followed by gastrointestinal disease [48,49]. In contrast, it has also been observed that respiratory diseases were the primary cause of yak calf mortality [15]. However, the authors further opined that, in the whole herd, mortality was highest due to gastrointestinal disorders such as diarrhea, followed by respiratory diseases. Consequently, a lower mortality rate was observed in calves aged more than three months of age [41,50]. The present findings suggest that environmental stress, lower physiological capacity, and nutritional deficiencies in neonatal calves may trigger vulnerability to enteric diseases, leading to death due to a high incidence of diarrhea in high-altitude yak production systems. Therefore, the predominance of diarrhoea observed in the present study likely reflects the combined effects of infectious agents and host susceptibility rather than a single causative pathogen. Future investigations incorporating laboratory diagnoses of enteric pathogens would help elucidate the specific causes of neonatal diarrhoea in yak calves.

Bovine respiratory disease (BRD) was the second-most frequently recorded cause of yak calf mortality in the present study. BRD is a multifactorial syndrome resulting from complex interactions among infectious agents, host immune status, and environmental factors [51]. However, because this investigation relied on retrospective farm records, the specific pathogens responsible for respiratory disease were not identified. In high-altitude yak production systems, sudden fluctuations in ambient temperature, cold stress, and humidity, as well as the compromised immunity in young calves, may further increase susceptibility to respiratory infections. The prevalence of respiratory diseases in the present study may be associated with the high-altitude environment and seasonal fluctuations in temperature, which could increase the susceptibility of young calves to respiratory infections [13,19]. As discussed earlier, low-birth-weight calves and calves born to primiparous dams may have poor thermoregulatory capacity, low energy reserves, and undeveloped neonatal vigor, which may increase susceptibility to respiratory diseases and contribute to the higher mortality observed in the present study. Subsequently, the relatively low death count due to tympany and other unknown factors demonstrated that these conditions were sporadic compared to the former diseases. Therefore, the present findings suggest that improving neonatal health through early detection and prompt treatment of diarrhea and BRD in affected calves may potentially enhance the survival of yak calves and, thereby, improve overall herd welfare. Since all animals included in the present study were maintained under a standardized management system at a single research farm, the influence of different feeding, housing, and breeding management practices on calf diseases could not be evaluated. Future multi-herd prospective studies comparing alternative nutritional and reproductive management strategies are warranted to determine their contribution to birth weight, neonatal health, and early-life survival in yak calves.

Although calf mortality is multifactorial, this study identified low birth weight and primiparous dam parity as the major risk factors for early-life mortality. Calf sex and birth season were not significantly associated with survival. The effects of maternal body weight and management practices could not be evaluated because these data were unavailable in the retrospective records and should be investigated in future prospective studies. The 13-year duration of the current study, performed at the same institution, accounts for potential temporal variations; the animals were maintained under standard operating procedures (SOPs) at a single research institute farm, naturally mitigating erratic year-to-year swings in management protocols compared to commercial multi-owner herds. Furthermore, the inclusion of birth year as a fixed effect and its validation via Schoenfeld residuals confirms that our primary findings regarding birth weight and dam parity are robust and independent of inter-annual trends.

The present study has some limitations as a retrospective study. Firstly, the analysis was limited to variables that have been routinely recorded on farm registry records. Secondly, there is a lack of information on colostrum intake, including timing and quantity, and colostrum intake is a vital factor influencing calf survival. Lastly, the severity of diseases was not evaluated, limiting further analysis of the contribution of health disorders to calf survival and mortality. Nevertheless, the 13-year duration of the cohort study using survival analysis provides solid proof of the main determinants contributing to early-life mortality in yak calves. Another methodological consideration is potential repeated-measures clustering, as individual dams may have produced multiple calves across the 13-year span. When accounting for potential repeated-measures clustering by incorporating individual Dam ID as a random effect, the frailty model yielded nearly identical results (HR ≈ 2.21 for low birth weight and HR ≈ 2.17 for primiparous dams, both remaining statistically significant at *p* < 0.01), confirming that within-dam correlation did not alter our primary conclusions. Although observations were treated independently in the primary fixed-effects model, supplementary analyses incorporating a dam-level shared frailty yielded comparable hazard ratios and consistent significance levels for birth weight and dam parity, confirming that within-dam clustering did not materially affect our primary conclusions. Another fact to be acknowledged was the absence of detailed information regarding the reasons for censoring. Consequently, censored calves could not be subdivided into those remaining alive, sold, or lost to follow-up. Although censoring was assumed to be non-informative, future prospective studies should systematically record the reasons for censoring to enable a more comprehensive assessment of potentially informative censoring.

## 5. Conclusions

This study identified low birth weight and dam parity as the major determinants associated with lower survival of yak calves during the first 90 days of life. In contrast, birth season and calf sex did not significantly influence survivability. These findings suggest that calf survival rates could be potentially increased by optimizing nutritional and reproductive management of primiparous dams, along with providing enhanced care for low-birth-weight calves during the neonatal period. Consequently, the utilization of survival analysis in the present study offers a valuable epidemiological insight for identifying high-risk calves and may facilitate the formulation of evidence-based management solutions to enhance productivity and sustainability in yak production systems. Furthermore, future research should incorporate standardized health records to elucidate how climate change at high altitudes interacts with these factors to determine long-term productivity.

## Figures and Tables

**Table 1:** Baseline characteristics of yak calves included in 90-day survival analysis from 2013 to 2025 (*n* = 428).

Variable (s)	Total (*n* = 428)	Survived (*n* = 358)	Died (*n* = 70)	*p* Value
Sex, n (%)				
Male	221 (51.6)	183 (51.1)	38 (54.3)	0.723
Female	207 (48.4)	175 (48.9)	32 (45.7)	
Birth weight category, n (%)				
Low BW (≤12 kg)	42 (9.8)	28 (7.8)	14 (20.0)	0.003
High BW (>12 kg)	386 (90.2)	330 (92.2)	56 (80.0)	
Parity category, n (%)				
Primiparous (parity = 1)	145 (33.9)	106 (29.6)	39 (55.7)	0.001
Multiparous (parity > 1)	283 (66.1)	252 (70.4)	31 (44.3)	
Birth season, n (%)				
Summer	53 (12.4)	46 (12.8)	7 (10.0)	0.658
Rainy	305 (71.3)	252 (70.4)	53 (75.7)	
Winter	70 (16.4)	60 (16.8)	10 (14.3)	

Data are presented as numbers (%). Survival status was assessed at 90 days of age. Calves surviving beyond 90 days were treated as censored observations. *p*-values were obtained using Pearson’s chi-square test.

**Table 2:** Multivariable Cox proportional hazards model identifying risk factors associated with yak calf mortality during the first 90 days of life (2013–2025).

Variable	Category	Hazard Ratio (HR)	95% *CI*	*p* Value
Birth weight category	High BW (>12 kg)	Ref	-	0.008
	Low BW (≤12 kg)	2.27	1.24–4.16	
Dam parity	Multiparous (>1)	-	-	0.003
	Primiparous (=1)	2.08	1.28–3.38	
Sex	Female	Ref	-	0.398
	Male	1.23	0.76–1.98	
Birth season	Rainy	Ref	-	
	Summer	0.86	0.39–1.90	0.701
	Winter	0.80	0.41–1.57	0.515

*HR* = hazard ratio; *CI* = confidence interval; Ref = reference. Mortality within the first 90 days was treated as an event, and calves beyond 90 days were considered censored. Reference categories were high BW (>12 kg), multiparous dams (parity > 1), female sex, and rainy season of birth.

**Table 3:** Global and variable-specific Schoenfeld residual tests evaluating the proportional hazards assumption of the multivariable Cox proportional hazards model.

Variable	χ^2^	df	*p*-Value
Sex	0.002	1	0.963
Birth season	0.096	2	0.953
Birth weight	0.021	1	0.884
Parity	8.899	7	0.260
Birth year	20.218	12	0.063
Global test	29.326	23	0.170

## Data Availability

The data are available upon reasonable request from the corresponding author.

## Supplementary Materials

The following supporting information can be downloaded at: https://www.mdpi.com/article/10.3390/ani16162614/s1, Table S1: Annual herd size and calf births during the study period (2013 to 2025); Table S2: Multivariable Cox proportional hazards frailty model (incorporating Dam ID as a random effect) evaluating risk factors associated with 90-day mortality in yak calves (n=428 observations, 70 events); Table S3: Kaplan–Meier estimates of overall survival probability at clinically relevant time points during the first 90 days of life in yak calves (*n* = 428); Table S4: Assessment of the birth weight × dam parity interaction in the Cox proportional hazards model.

## Abbreviations

The following abbreviations are used in this manuscript:

QTP	Qinghai–Tibetan Plateau
FTPI	Failure of Passive Immunity Transfer
BW	Birth Weight
ICAR	Indian Council of Agricultural Research
K-M	Kaplan–Meier
BRD	Bovine Respiratory Disease
