# Supplementary material for: Unravelling the Drivers of Early-Life Mortality in Yak Calves: A Retrospective Cohort Study Using Survival Analysis
Source: Animals (Basel). 2026 Aug 20;16(16):2614. doi: 10.3390/ani16162614 (PMC13509976; doi:10.3390/ani16162614)
Supplement: Supplementary file 1 [file animals-16-02614-s001.zip › animals-4468038-supplementary.pdf]

Supplementary Table S1. Annual herd size and calf births during the study period (2013 to 2025).

Year	Total herd size	Breeding females	Calves born
2013	194	124	18
2014	128	73	13
2015	128	83	20
2016	138	96	38
2017	174	110	36
2018	199	122	33
2019	199	122	48
2020	200	136	45
2021	198	135	49
2022	187	130	24
2023	151	110	32
2024	148	105	30
2025	168	113	42

Supplementary Table S2. Multivariable Cox proportional hazards frailty model (incorporating Dam ID as a random effect) evaluating risk factors associated with 90-day mortality in yak calves (n = 428 observations, 70 events).

Variable / Category	Coefficient (β)	Hazard Ratio (*HR*)	Standard Error (SE)	z-value	*p*-value
**Birth Weight**					
High BW (> 12 kg) [Ref]	—	1.00	—	—	—
Low BW (≤ 12 kg)	0.7945	2.21	0.2929	2.71	**0.0067**
**Dam Parity**					
Multiparous (> 1) [Ref]	—	1.00	—	—	—
Primiparous (= 1)	0.7733	2.17	0.2544	3.04	**0.0024**
**Sex**					
Female [Ref]	—	1.00	—	—	—
Male	0.1568	1.17	0.2548	0.62	0.5384
**Birth Season**					
Rainy [Ref]	—	1.00	—	—	—

**Variable / Category**	**Coefficient (β)**	**Hazard Ratio (*HR*)**	**Standard Error (SE)**	**z-value**	***p*-value**
Summer	-0.1643	0.85	0.4520	-0.36	0.7163
Winter	-0.1799	0.84	0.3914	-0.46	0.6459
**Birth Year** *(Fixed effects)*					
Year 2013 [Ref]	—	1.00	—	—	—
Year 2014	-0.1877	0.83	0.7670	-0.24	0.8067
Year 2015	-0.7570	0.47	0.7718	-0.98	0.3266
Year 2016	-0.3621	0.70	0.6201	-0.58	0.5593
Year 2017	-1.5819	0.21	0.8537	-1.85	0.0639
Year 2018	-1.7083	0.18	0.8506	-2.01	**0.0446**
Year 2019	-0.2756	0.76	0.5479	-0.50	0.6149
Year 2020	-1.5078	0.22	0.6819	-2.21	**0.0270**
Year 2021	-0.2626	0.77	0.5435	-0.48	0.6289
Year 2022	-0.2554	0.77	0.6748	-0.38	0.7051
Year 2023	-0.9611	0.38	0.7764	-1.24	0.2158
Year 2024	-0.3280	0.72	0.5853	-0.56	0.5752
Year 2025	-1.2988	0.27	0.6858	-1.89	0.0582
**Random Effect (Dam ID)**	*Standard Deviation*	*Variance*			
Intercept	0.5433	0.2952			

Note: Models fitted using the coxme package in R. Reference categories are indicated with [Ref]. Bold values indicate statistical significance ($P < 0.05$).

Supplementary Table S3. Kaplan–Meier estimates of overall survival probability at clinically relevant time points during the first 90 days of life in yak calves (n = 428).

Time (days)	Survival probability	95% *CI*	Number at risk	Events at time points
7	0.974	0.959–0.989	418	11
30	0.949	0.928–0.970	406	11
60	0.921	0.895–0.947	395	12
90	0.836	0.802–0.872	376	36

Supplementary Table S4. Assessment of the Birth weight × Dam parity interaction in the Cox proportional hazards model

Interaction	Coefficient (β)	Hazard Ratio (*HR*)	95% *CI*	z-value	p-value
Birth weight (Low BW) × Primiparous dam	0.884	2.42	0.55–10.65	1.17	0.242
